# Supplementary material for: The Lipidomic Profile Is Associated with the Dietary Pattern in Subjects with and without Diabetes Mellitus from a Mediterranean Area
Source: Nutrients. 2024 Jun 8;16(12):1805. doi: 10.3390/nu16121805 (PMC11206394; doi:10.3390/nu16121805)
Supplement: Supplementary file 1 [file nutrients-16-01805-s001.zip › nutrients-3026771-supplementary.pdf]

Supplementary Material

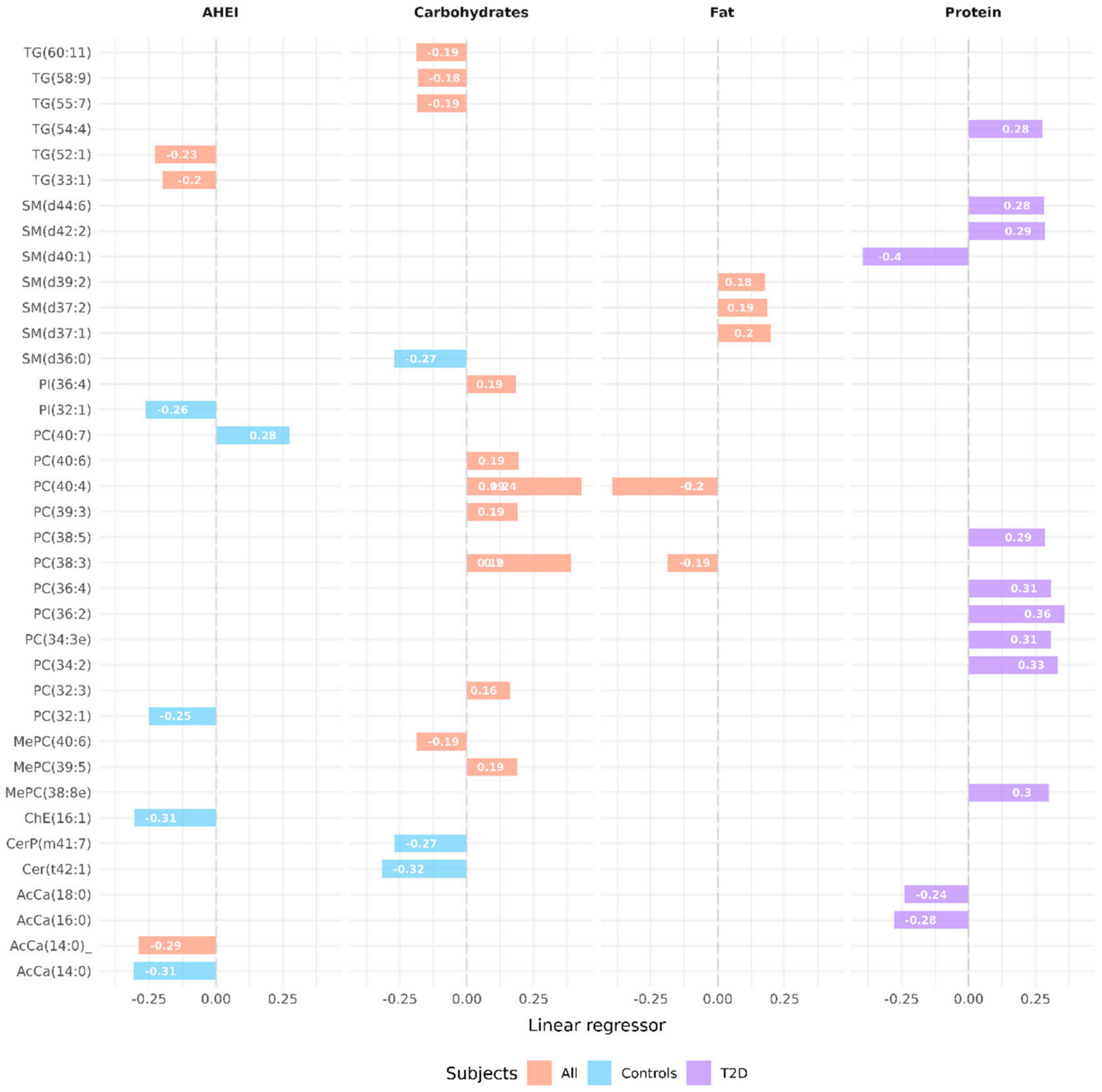

Supplementary Figure S1. Complete density plot for aHEI and aMED scores. aMED, alternate Mediterranean Diet score; aHEI, alternate Healthy Eating Index; T1D, type 1 diabetes; T2D, type 2 diabetes

Supplementary Table S1. Interaction between diabetes and aHEI

Lipids	Class	Beta	q-value	Ionization	Confirmed
LPC(18:2e)+H	LPC	-0.398	0.00541	Positive	Yes
LPC(18:1e)+H	LPC	-0.429	0.00216	Positive	Yes
PC(40:3e)+H	PC	-0.539	0.00348	Positive	No
PC(32:1)+H	PC	0.420	0.03365	Positive	No

Lipid annotation; class of the lipid; q-value, list of corrected p-values for each analysis where the lipid is significant; Beta, list of linear regressors for each significant analysis; Ionization, acquisition mode; Confirmed, annotation through LipidSearch.

LPC, lysophosphatidylcholines; PC, phosphatidylcholine
